# Impact of declared wildfire disasters on survival of lung cancer patients undergoing radiation

**DOI:** 10.1007/s10552-024-01949-2

**Published:** 2025-01-09

**Authors:** Katie E. Lichter, Bria Larson, Meghana Pagadala, Osama Mohamad, Leticia Nogueira

**Affiliations:** 1 Department of Radiation Oncology, University of California San Francisco, San Francisco, CA USA; 2 School of Public Health, University of California Berkeley, Berkeley, CA USA; 3 Department of Medicine, Memorial Sloan Kettering Cancer Center, New York, USA; 4 Department of Radiation Oncology, MD Anderson, Houston, TX USA; 5 Surveillance and Health Equity Science, American Cancer Society, Kennesaw, GA USA; 6 Helen Diller Family Comprehensive Cancer Center, University of California San Francisco, San Francisco, CA USA

**Keywords:** Wildfires, Radiotherapy, Oncology, Lung cancer, Disasters, Survival

## Abstract

**Purpose:**

Oncological treatments, such as radiotherapy, which requires consistent electricity, the presence of specialized clinical teams, and daily patient access to treatment facilities, are frequently disrupted by extreme weather events, posing several health hazards to patients. This study explores the association between declared wildfire disasters during radiotherapy and overall survival among patients with non-small cell lung cancer (NSCLC).

**Methods:**

The study population consisted of 202,935 adults with inoperable Stage III NSCLC, who initiated radiotherapy from 2004 through 2019. Exposure was defined as a wildfire disaster declaration in the county of the treatment facility within 12 weeks of initiating radiotherapy. Overall survival was defined as the interval (months) between age at diagnosis and age at death, date of last contact, or study end. Cox proportional hazards was used to estimate crude and adjusted hazard ratios and 95% confidence intervals with inverse probability weighting.

**Results:**

Patients exposed to a wildfire disaster declaration during radiation treatment had worse overall survival (HR, 1.03; 95% CI 1.00–1.06; *p* = 0.02), compared to unexposed patients in adjusted models.

**Conclusion:**

Exposure to a wildfire disaster during radiotherapy is associated with worse overall survival among patients with stage III non-operable NSCLC. This finding underscores the critical need for developing adaptation strategies within the healthcare sector, especially in oncology.

## Background

Climate change is one of the strongest factors contributing to increasing wildfire activity in the US [1–3]. Climate-driven disasters, such as wildfires, can damage medical infrastructure (e.g., facilities, equipment), compromise transportation routes, interrupt supply chains, and frequently result in disruptions in access to cancer care [4].

Oncological treatments such as radiotherapy, which requires consistent electricity, daily patient access to the treating facility, and the presence of specialized teams, are particularly vulnerable to disruptions caused by climate-driven extreme weather events [5]. One previous study found 100% of radiation oncology clinics in California were within 25 miles of a wildfire between 2017 and 2022, most of which (61%) experienced disruptions in services including canceling scheduled treatments [6].

Disruptions in access to radiation treatment are especially concerning for patients diagnosed with aggressive tumors, such as locally advanced non-small cell lung cancer (NSCLC) [7], for whom daily radiation treatment over several weeks is critical to achieving optimal outcomes [8]. Importantly, NSCLC remains the leading cause of cancer-related deaths worldwide [9], with an overall 5-year survival rate of 25% despite advances in therapy [10]. For early-stage disease, surgery is usually curative, while a combination of chemotherapy and radiation therapy, and more recently immunotherapy, are the recommended treatment strategies for advanced disease [11]. Risk factors for mortality include older age, comorbidities, and more advanced disease [11]. Further, even minor delays in radiation therapy—such as a treatment completion time prolonged by just 2 days—are associated with reduced overall survival [12–15].

Previous research found that exposure to hurricane disasters during radiotherapy was associated with worse survival among patients with NSCLC [16]. However, the impact of climate-driven disasters on cancer outcomes remains largely unrecognized and unaddressed, and there are still no guidelines for addressing the detrimental health consequences associated with exposure to disasters during cancer treatment [17]. Better characterizing the association between exposure to climate-driven disasters and mortality is fundamental for informing future research efforts aimed at developing disaster preparedness and response strategies aimed at better protecting the health and safety of patients undergoing cancer treatment in the era of climate change. Therefore, this study builds on prior research and explores the association between declared wildfire disasters during radiotherapy and overall survival among patients diagnosed with inoperable locally advanced NSCLC.

## Methods

Individuals aged 18 years and older diagnosed with Stage III NSCLC, who did not receive surgical treatment and initiated definitive radiotherapy from 2004 to 2019 were identified in the National Cancer Database (NCDB), a hospital-based cancer registry co-sponsored by the American College of Surgeons and the American Cancer Society. [18] The NCDB follows the North America Association of Central Cancer Registries (NAACCR) guidelines for cancer data abstraction and processing, which ensures uniform registry processes and ensures data quality standards [19]. Therefore, cases were selected using established ICD-O-3 topology and histology codes for NSCLC [20]. Exposure was defined as a Federal Emergency Management Agency (FEMA) wildfire disaster declaration in the county of the treatment facility within 12 weeks of initiating radiotherapy, as this period reflects the typical duration of treatment for locally advanced NSCLC, including potential delays or interruptions [8, 21]. Time-varying inverse probability weights were generated using logistic regression including geographic region, age, sex, chemotherapy initiation, and month of follow-up to correct for immortal time bias because exposed patients had guaranteed survival between date when radiotherapy started and date of exposure [22]. Geographic region was categorized into Northeast (CT, MA, ME, NH, NJ, NY, PA, RI, VT), Midwest (IA, IL, IN, KS, MN, MO, MI, NE, ND, OH, SD, WI), South (AL, AR, DE, DC, FL, GA, LA, KY, MD, MS, NC, SC, OK, TN, TX, VA, WV), West (AZ, CA, CO, ID, MT, NM, NV, OR, UT, WA, WY) following the US Census Bureau definition [23]. Patients exposed to coastal storm, flood, hurricane, severe ice storm, severe storm, snowstorm, tornado, tropical depression, tropical storm, and winter storm disasters were censored at the date when the disaster was declared.

Overall survival was defined as the interval (months) between age at diagnosis and age at death, last known contact, or study end (December 31, 2019), whichever occurred first. A Cox proportional hazards model was used to estimate crude and adjusted hazard ratios (HRs) and 95% confidence intervals (CIs) with inverse probability weighting. Adjusted models included sex, geographic region, health insurance type, comorbidities defined using the modified Charlson and Deyo comorbidity index for cancer patients (categorized into 0, 1, or ≥ 2) [24], lymph node involvement, type of facility, and concomitant chemotherapy. These variables were selected as potential confounders for the association of exposure to wildfire disaster during radiotherapy and cancer outcomes using qualitative a priori subject matter knowledge [25].

Descriptive statistics included self-identified race and ethnicity to show the diversity of the study population. We chose not to include race and ethnicity in the adjusted models to avoid incorrectly assigning race (a social construct that should only be used as a proxy for exposure to racism) as a risk factor [26].

The institutional review board of the Morehouse School of Medicine granted an exemption for this study in accordance with 45 CFR §46.102(f).

## Results

Of a total of 202,935 individuals included in the study, 1,513 (0.75%) were exposed to a wildfire disaster within 12 weeks of radiation treatment start date. Mean age at radiotherapy start date was 67.9 years and the majority of patients were male (54.6%). Exposure to a wildfire disaster during radiotherapy was most common among patients residing in the West region (73.4%), followed by the South (25.8%). No patients were exposed to > 1 wildfire disaster within 12 weeks of initiating treatment. The median follow-up was 14 months. In the exposed group, the total number of deaths was 1,248 and mean survival time was 24.8 months. In the unexposed group, the total number of deaths was 162,366 and mean survival time was 23.8 months. Although the association was not significant in crude models (HR 0.98; 95% CI 0.96–10.3; *p* = 0.08), patients exposed to a wildfire disaster declaration during radiation treatment had worse overall survival (HR, 1.03; 95% CI 1.00–1.06; *p* = 0.02) than unexposed patients in adjusted models.

## Discussion

Exposure to a wildfire disaster during radiotherapy is associated with worse overall survival among patients diagnosed with stage III non-operable NSCLC. The results of the present study align with the previous study reporting worse overall survival among patients undergoing radiotherapy for non-operable locally-advanced NSCLC [16]. Hurricane disasters potentially result in more dramatic disruptions in radiotherapy care, as previously reported [27], while wildfires are potentially more common, especially in the West region of the US [6]. Moreover, the dynamic and frequently compounding hazards posed by wildfires, including air contaminated with smoke, water and soil contamination with byproducts of burned vegetation and human-made products, and the cognitive and physical challenges of following evacuation orders [28], interact with the physical and psychological challenges experienced by patients diagnosed with NSCLC [22], potentially increasing vulnerability to wildfire disasters hazards in this population (Tables 1 and 2).

**Table 1:** Demographic and clinical characteristics of patients with stage III non-operable NSCLC undergoing radiotherapy 2004–2019

		Overall	Exposed	Unexposed
*n*		202,935	1513	201,422
Age at radiotherapy start date, mean (SD)		67.9 (10.3)	68.5 (10.7)	67.8 (10.3)
Sex, *n* (%)	Male	110,724 (54.6)	790 (52.2)	109,934 (54.6)
	Female	92,171 (45.4)	722 (47.8)	91,449 (45.4)
Race/ethnicity, *n* (%)	Non-Hispanic White	167,868 (83.2)	1165 (77.2)	166,703 (83.2)
	Hispanic	4502 (2.2)	120 (7.9)	4382 (2.2)
	Non-Hispanic Black	24,996 (12.4)	111 (7.4)	24,885 (12.4)
	Asian & Pacific Islander	3275 (1.6)	102 (6.8)	3173 (1.6)
	Other	1137 (0.6)	12 (0.8)	1125 (0.6)
Facility Geographic Region, *n* (%)	Northeast	40,705 (20.1)	13 (0.9)	40,692 (20.2)
	Midwest	62,268 (30.7)	0 (0.0)	62,268 (30.7)
	South	77,341 (38.1)	390 (25.8)	76,951 (38.2)
	West	22,621 (11.1)	1110 (73.4)	21,511 (10.7)
Concomitant chemotherapy, *n* (%)	No	152,445 (75.1)	1123 (74.2)	151,322 (75.1)
	Yes	50,490 (24.9)	390 (25.8)	50,100 (24.9)
Lymph node involvement, *n* (%)	No	159,083 (78.4)	1188 (78.5)	151,322 (78.4)
	Yes	43,852 (21.6)	325 (21.5)	43,527 (21.6)
Comorbidity, *n* (%)	0	123,517 (60.9)	1046 (69.1)	122,471 (60.8)
	1	51,038 (25.1)	317 (21.0)	50,721 (25.2)
	2 or more	28,380 (14.0)	150 (9.9)	28,230 (14.0)
Facility type (%)	National Cancer Institute–designated	18,327 (9.0)	169 (11.2)	18,158 (9.0)
	Comprehensive	78,335 (38.6)	582 (38.5)	77,753 (38.6)
	Teaching	37,167 (18.3)	179 (11.8)	36,988 (18.4)
	Other	69,106 (34.1)	583 (38.5)	68,523 (34.0)
Median income quintile, $ (%)	< 36,000	36,016 (17.8)	191 (12.8)	35,825 (17.9)
	36,000–43,999	43,300 (21.5)	256 (17.2)	43,044 (21.5)
	44,000–52,999	43,239 (21.4)	307 (20.6)	42,932 (21.4)
	53,000–68,999	43,968 (21.8)	364 (24.4)	43,604 (21.8)
	> = 69,000	36,292 (17.5)	372 (25.0)	34,920 (17.4)

*Data Sources*: Demographic data from the National Cancer Data Base (NCDB). Exposure data from Federal Emergency Management Agency (FEMA)

Other includes all Indigenous Populations on the Western hemisphere and the option “other”, which was provided to patients during data collection

**Table 2:** Mortality risk for patients exposed to a wildfire while receiving radiotherapy for stage III non-operable NSCLC, National Cancer Database (2004–2019)

Crude and adjusted estimates of hazard ratio			
	HR	95% CI	*p*-value
Crude	0.98	(0.96–1.03)	0.08
Adjusted	1.03	(1.00–1.06)	0.02

Strengths of the study include the large national sample of patients diagnosed with NSCLC (over 70%) [18], in all regions of the US, with detailed clinical and demographic information collected according to established cancer registry standards [19]. Limitations include lack of information on date and reason for treatment breaks, disaster-related issues (e.g., displacement, worsening mental health, financial hardship), patient prognostic factors (smoking history, performance status), and inability to account for within-county variability in exposure to wildfire hazards as FEMA presidential disasters are declared at the county-level.

The findings underscore the critical need for developing adaptation strategies within the healthcare sector, especially in oncology, to mitigate the adverse effects of climate-driven disasters on vulnerable patient populations [17, 29]. Future studies should evaluate whether characteristics of population subgroups known to be disproportionately impacted by worse health outcomes after NSCLC diagnosis modify the association between exposure to climate-driven disasters during cancer treatment and survival, as well as the potential of disaster recovery strategies aimed at modifiable factors such as altered radiation treatment schedule, post-disaster housing, mental health, diet, physical activity, and/or social support to alleviate the detrimental health consequences associated with exposure to climate-driven disasters.

## Data Availability

Federal Emergency Management Agency (FEMA) data on presidential disaster declarations is publicly available at: https://www.fema.gov/openfema-data-page/disaster-declarations-summaries-v2. National Cancer Database (NCDB) data is available to researchers affiliated with Commission on Cancer accredited facilities in compliance with the Data Use Agreement: https://www.facs.org/quality-programs/cancer-programs/national-cancer-database/puf/
